# PromoterAtlas: decoding regulatory sequences across Gammaproteobacteria using a transformer model

**DOI:** 10.1038/s41467-026-72837-3

**Published:** 2026-05-15

**Authors:** Lucas Coppens, Rodrigo Ledesma-Amaro

**Affiliations:** 1 London Biofoundry, Translation and Innovation Hub, Imperial College White City Campus, London, UK; 2 Department of Infectious Disease, Imperial College London, London, UK; 3 Department of Bioengineering, Imperial College London, London, UK; 4 Bezos Centre for Sustainable Protein, Imperial College London, London, UK; 5 UKRI Engineering Biology Mission Hub on Microbial Food, Imperial College London, London, UK

**Keywords:** Machine learning, Bacterial genomics, Gene expression, Gene regulation, Sequence annotation

## Abstract

Recent advances in deep learning, particularly transformer architectures, have improved computational approaches for biological sequence analysis. Despite these advances, computational models for bacterial promoter prediction have remained limited by small datasets, species-specific training, and binary classification approaches rather than comprehensive annotation frameworks. We present PromoterAtlas, a 1.8 M parameter transformer model trained on 9 M regulatory sequences from 3371 gammaproteobacterial species. The model demonstrates recognition of various regulatory elements across different species, including ribosomal binding sites, various types of bacterial promoters, transcription factor binding sites, and terminators. Using this model, we developed a whole-genome promoter annotation tool for Gammaproteobacteria, with various levels of validation that support the predictions of promoters associated with different sigma (σ) factors. Furthermore, we show that the model embeddings reflect cross-species evolutionary relationships, clustering promoters by σ factor identity rather than species-specific sequence features. Finally, we show that model embeddings encode regulatory sequence information that enables effective prediction of transcription and translation levels. PromoterAtlas can contribute to our understanding of and ability to engineer bacterial regulatory sequences with potential applications in bacterial biology, synthetic biology, and comparative genomics.

## Abstract (web summary)

Coppens & Ledesma-Amaro present a transformer-based computational model trained on 9 million bacterial sequences from over 3,000 species, which enables automated identification of diverse promoter types across *Gammaproteobacteria* as well as prediction of gene expression levels.

## Introduction

Sequence-based transformer models have recently revolutionised bioinformatics. In the genomic domain, massive models such as Enformer^1^, Nucleotide Transformer^2^, Evo 2^3^, and DNABert-2^4^ have demonstrated unprecedented capabilities in capturing complex sequence patterns and functional relationships across DNA. In the protein domain, the model ESM-2 and the associated ESMFold in particular have shown that purely sequence-based models can be used to predict structural properties with remarkable accuracy, enabling a breakthrough in protein folding^5^. These models leverage self-attention mechanisms to identify long-range patterns between nucleotides or amino acids. By learning contextual representations directly from genomic or protein sequences, transformer architectures have managed to overcome the limitations of previous architectures and approaches that relied heavily on hand-crafted features or position-specific scoring matrices.

Bacterial promoter prediction has been addressed through various computational approaches, including position weight matrices (PWMs), hidden Markov models, support vector machines, convolutional neural networks, recurrent neural networks, and most recently transformer architectures^6–15^. While deep learning approaches have demonstrated improved performance over traditional PWMs and HMMs in benchmark evaluations, they remain constrained by several fundamental limitations that have prevented their translation into practical genome annotation tools^16,17^. The scarcity of experimentally validated promoters creates a bottleneck in training data, with most models relying on small sets of positive labels that inadequately represent the diversity of bacterial regulatory elements. Furthermore, bacterial promoter models are often trained on data limited to single species, most often *Escherichia coli*^6,11,13^ and other representative species such as *Bacillus subtilis*^18^ and *Pseudomonas aeruginosa*^9,10^, limiting both dataset size and model generalisability. Perhaps most critically, existing approaches typically focus on classifying isolated sequences rather than providing a comprehensive annotation framework. This methodological gap has left automated whole-genome promoter annotation an unresolved challenge, hampering our ability to rapidly annotate newly sequenced bacterial genomes and characterise regulatory landscapes. This annotation deficit is exemplified by the near-complete absence of promoter annotations in public databases: among the GenBank files of 3371 gammaproteobacterial genomes used for training in this study, we found only 263 annotated promoter features compared to nearly 8.5 million annotated coding sequences. This imbalance underscores the critical need for scalable automated annotation tools.

We devised an approach incorporating multiple solutions to address current constraints in bacterial promoter prediction. First, by leveraging unsupervised masked-token learning, we circumvent the limitation of scarce labelled data, allowing the model to discover intrinsic patterns within sequences without requiring explicit promoter annotations. We strategically create a focused dataset consisting of regions spanning 200 nucleotides upstream of coding sequences, thereby creating an enrichment for relevant regulatory signals while reducing noise from irrelevant genomic regions. Finally, we extracted data from a wide range of sequenced genomes of species across the clade of Gammaproteobacteria, capitalising on presumed evolutionary conservation of promoter architecture within this phylogenetic group. The Gammaproteobacteria is one of the most extensively studied bacterial clades, spanning approximately 250 genera and containing diverse medically and biotechnologically relevant species such as *E. coli*, *P. aeruginosa*, *Pseudomonas putida*, *Salmonella enterica*, *Vibrio cholerae*, *Vibrio natriegens*, *Yersinia pestis*, and *Klebsiella pneumoniae*. This taxonomic breadth introduces sufficient sequence diversity to capture variations in promoter structure while maintaining conservation of core regulatory mechanisms.

In this paper, we present PromoterAtlas, a 1.8 million parameter model trained on approximately 9 million regulatory sequences from 3371 diverse gammaproteobacterial species. We show that visualisation of pre-trained base model logits enables identification of functional regulatory elements, including promoters, ribosomal binding sites (RBS), transcription factor binding sites, and terminators. We extend this capability by developing a segmentation head that transforms embeddings from the base model into annotations for genome-wide identification of promoters across five σ factors in Gammaproteobacteria, with validation through ChIP-seq data comparison, analysis of predicted promoter distributions, and gene ontology enrichment studies. Additionally, we demonstrate that the network embeddings reflect evolutionary relationships between different promoter types across phylogenetically distant Gammaproteobacterial species, as revealed through analyses of its embedding space. Finally, we show that PromoterAtlas embeddings can be leveraged for sequence-based prediction of gene expression at both transcription and translation levels.

## Results

**Fig. 1:**
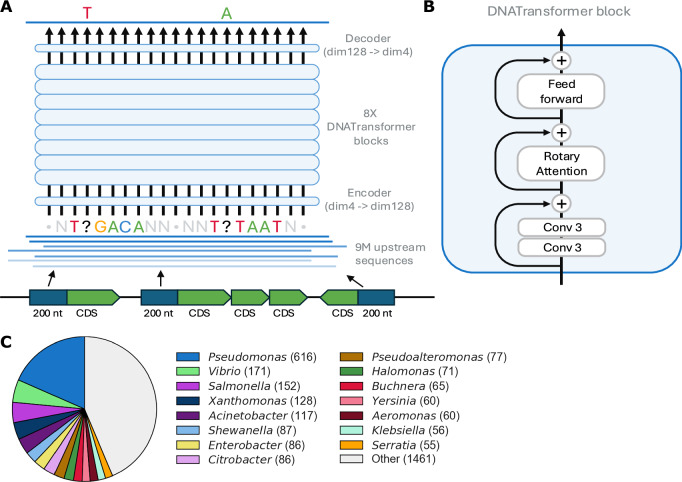
Pre-training a base model on regulatory sequences across the clade of the Gammaproteobacteria. **A** Overview of the pre-training workflow. 200 nucleotide sequences upstream of all coding sequences (CDS) are extracted from the genomes of the Gammaproteobacteria in the training set. During training, positions are randomly masked, and the transformer model is optimised to predict the missing tokens from the sequence’s context, encouraging it to learn relevant sequence features such as promoter motifs (e.g., TTGACA-TATAAT). **B** Overview of the DNATransformer block architecture used in PromoterAtlas. **C** Overview of Gammaproteobacterial genera included in the data set used to pre-train PromoterAtlas.

PromoterAtlas-base was obtained by masked-token prediction training on approximately 9 million 200-nucleotide sequences upstream of coding sequences from 3371 gammaproteobacterial species using masked token prediction. The DNATransformer architecture combines convolutional filters for capturing local sequence patterns with rotary attention layers for detecting long-range dependencies (Fig. 1). The machine learning task was masked token prediction on zero-masked positions, encouraging the model to learn regularly recurring patterns such as promoter motifs, as masked nucleotides occurring within such motifs are more predictable than nucleotides at less biologically relevant positions. Training converged after 264 epochs, showing strong generalisation between training and validation datasets (Supplementary Fig. S1).

### Recognition of regulatory features

**Fig. 2:**
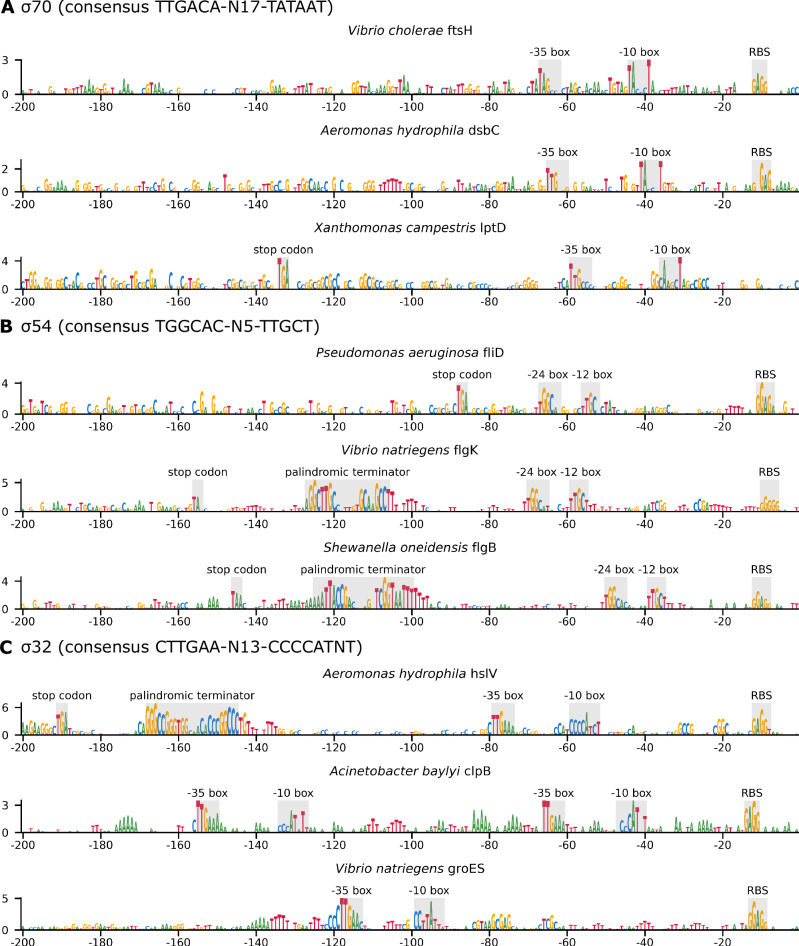
Logit plots of regulatory sequences upstream of coding sequences. The plots show recognition of a wide variety of motifs in various Gammaproteobacteria including RBS, motifs of promoters belonging to at least five σ factors, stop codons, and terminators. Coordinates are relative to the first base of the following gene’s start codon. The manually added annotations show what features are being recognised by PromoterAtlas in the areas with high per-nucleotide logits **A** logit plots showing recognition of σ70 promoter motifs **B** logit plots showing recognition of σ54 promoter motifs **C** logit plots showing recognition of σ32 promoter motifs.

**Fig. 3:**
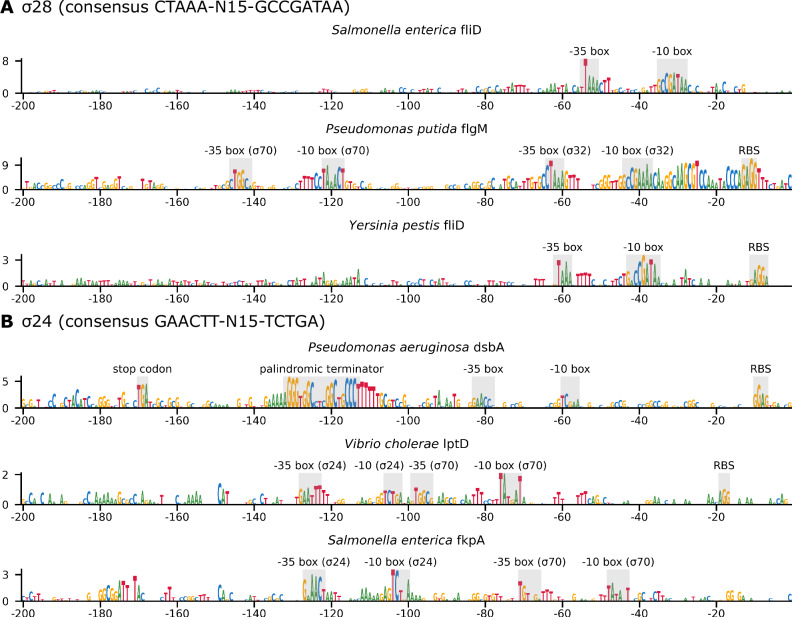
Logit plots of regulatory sequences upstream of coding sequences. The plots show recognition of a wide variety of motifs in various Gammaproteobacteria, including RBS, motifs of promoters belonging to at least five σ factors, stop codons, and terminators. Coordinates are relative to the first base of the following gene’s start codon. The manually added annotations show what features are being recognised by PromoterAtlas in the areas with high per-nucleotide logits **A** logit plots showing recognition of σ28 promoter motifs **B** logit plots showing recognition of σ24 promoter motifs.

Plotting the per-base pre-softmax logits after the linear projection in the decoder revealed the sequence patterns recognised by the pre-trained model in individual sequences (Figs. 2, 3). This visualisation method highlighted a wide variety of recognisable sequence features, including promoters corresponding to at least 5 σ factors, as well as RBS. Palindromic terminators were also often recognised by the model despite only sequences upstream of coding sequences having been included in the training dataset. This reflects the compact organisation of bacterial genomes: short intergenic distances cause 200 nucleotide upstream regions to frequently include terminators from preceding genes. Similarly, the logit plots showed that PromoterAtlas recognised stop codons when the preceding gene overlapped with the analysed 200 nucleotide upstream sequences. We also found that the model showed recognition of known motifs of various transcription factor binding sites, including motifs for CRP, ntrC, luxR, and FNR (Supplementary Fig. S2).

In some cases, particularly for upstream regions of genes known to have high expression levels in bacteria, large logit values along long stretches of the analysed sequences impeded clear distinction between promoter motifs and surrounding nucleotides (Supplementary Fig. S3). In those cases, plotting the transformer’s attention maps was helpful in revealing the location of the promoter motifs. These attention maps also revealed that the model focuses especially on the final position in the −10 box of the promoters.

### Whole genome promoter motif annotation

**Fig. 4:**
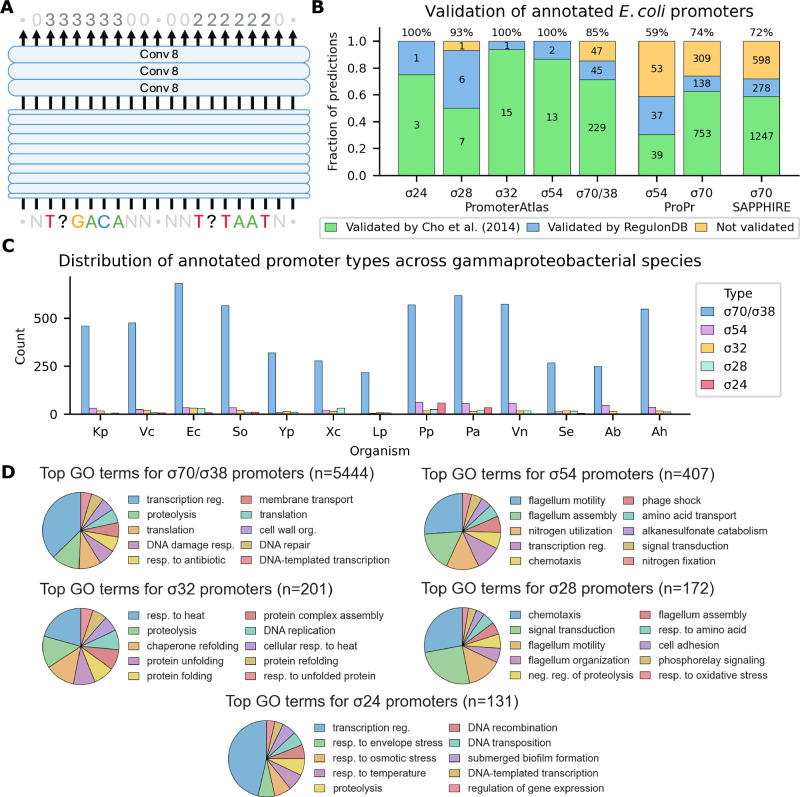
A segmentation head leveraging the PromoterAtlas-base embedding enables automated genome-wide promoter annotation for 5 σ factors. **A** A segmentation head consisting of three convolutional filters of width 10 allows to transform the PromoterAtlas-base final layer embeddings into per-position discrete outputs that can be used for automated sequence annotation. **B** Validation of PromoterAtlas (PA) annotations, ProPr54^23^, ProPr70(2014)^21^, and SAPPHIRE (SP)^9^ σ70 promoter predictions in *E. coli* by Cho et al.^21^ and RegulonDB^22^. **C** The number of automatically annotated promoter motifs for each σ factor in selected Gammaproteobacteria: Kp (*K. pneumonia*), Vc (*V. cholerae*), So (*Shewanella oneidensis)*, Yp (*Y. pestis*), Xc (*Xanthomonas campestris*), Lp (*L. pneumophila*), Pp (*P. putida*), Pa (*P. aeruginosa*), Vn (*V. natriegens*), Se (*S. enterica*), Ab (*Acinetobacter baylyli*), Ec (*E. coli*), Ah (*Aeromonas hydrophila*). **D** The top 10 gene ontology terms associated with the genes following predicted annotations of the different σ factors in (**C**). The overall number of annotated promoter-gene pairs for each sigma factor is included between parenthesis.

Since the PromoterAtlas-base logit plots of Gammaproteobacterial regulatory sequences frequently highlighted sequence features that we could easily recognise as known promoter patterns (Figs. 2, 3), we developed a segmentation-based approach to automate their identification and annotation, called PromoterAtlas-annotation (Fig. 4A). In this approach, a segmentation head is trained to transform the PromoterAtlas-base final-layer embeddings representing input sequences into a discrete signal indicating the presence of promoter elements. This method mirrors successes in transfer learning in image classification using vision transformers, where a handful of examples are often sufficient to achieve excellent performance due to the richness of pretrained transformer embeddings^19,20^. The segmentation head was trained using 318 manually curated sequences containing clearly identifiable promoter motifs from the logit plots, representing five σ factor classes across multiple species (σ70/σ38: *n* = 147; σ54: *n* = 50; σ32: *n* = 76; σ28: *n* = 27; σ24: *n* = 18). No *E. coli* promoters were included in the segmentation head training data, as *E. coli* was the only organism for which we found genome-wide ChIP-seq data for various σ factor binding sites^21^, presenting an opportunity to validate the segmentation tool on out-of-training data.

Following application of the PromoterAtlas-annotation segmentation head, the resulting post-softmax logits require conversion to discrete annotated features for automated sequence annotation. Two parameters determining this conversion were optimised: the minimum of consecutive segmentation-classified nucleotides required to constitute a valid promoter feature, as well as enforcement of co-occurrence rules mandating the presence of both canonical binding elements (e.g., −35 and −10 boxes for σ70, or −24 and −12 boxes for σ54). A sensitivity analysis on these two parameters identified a threshold of 3 consecutive classified bases combined with co-occurrence enforcement as the optimal setting, yielding maximal precision against the Cho et al.^21^ ChIP-seq validation data (Supplementary Fig. S4). For the following analyses, we employed this precision-optimised configuration over recall, reasoning that genome-wide annotation applications benefit from fewer high-confidence predictions rather than many potentially uncertain annotations.

Figure 4B shows the results of the different promoter types identified in the *E. coli* genome by the high-precision annotation tool, and their respective fractions, which could be validated by either the Cho et al.^21^ ChIP-seq data or the RegulonDB database^22^. Validation rates were high across all σ factor classes: σ24 (100%), σ28 (100%), σ32 (100%), σ54 (100%), and σ70 (85%). Crucially, for the single predicted but unvalidated σ28 promoter, as well as many examined unvalidated σ70 promoters, we found that the logit plots exhibited clear promoter motifs matching the binding sites of these σ factors (Supplementary Fig. S5). We therefore deemed these predictions plausible, estimating real precision would be higher still.

To contextualise these results, we also evaluated PromoterAtlas-annotation against existing tools by comparing its promoter predictions with those from ProPr54^23^ (σ54), ProPr^24^ (σ70), and SAPPHIRE^9^ (σ70). While more widely cited tools like BPROM^6^ and CNNPromoter^11^ exist, their web interfaces permit analysis of only one DNA sequence at a time and therefore do not support high-throughput analysis of regulatory sequences extracted from GenBank files, limiting their utility for genome-scale annotation. When applying the same ChIP-seq and RegulonDB validation criteria, both ProPr and SAPPHIRE predicted substantially more promoters but with lower precision (Fig. 4B): ProPr54, ProPr70, and SAPPHIRE achieved validation rates of 58%, 74%, and 71%, respectively. In contrast, PromoterAtlas-annotation achieved an overall 88% validation rate. This high precision across σ factor classes validates our parameter optimisation strategy.

We subsequently used the segmentation model to rapidly predict and annotate hundreds of promoter motifs in a variety of medically and biotechnologically relevant gammaproteobacterial species (Fig. 4C). PromoterAtlas-annotation identifies varying numbers of promoters depending on the species, with the smallest number of promoters predicted in *Legionella pneumophila* (238), and the largest number of promoters detected in *P. aeruginosa* (743). This likely reflects variability in promoter motif conservation, though high *Pseudomonas* representation in training data may contribute to the model’s enhanced pattern recognition within the *Pseudomonas* genus. The relative abundances of predicted promoters across different σ factors also align with biological expectations rather than training set composition, providing an additional layer of validation. For instance, the proportion of predicted σ70/σ38:σ54 promoters is roughly 12:1, reflecting natural bacterial distributions more closely than the 3:1 ratio present in the training data. Similar divergences toward known biological distributions are evident across the other σ factors, indicating that PromoterAtlas-annotation recognises genuine promoter patterns rather than merely reproducing training set biases.

Finally, we examined whether the genes downstream of the annotated promoter motifs correspond to biological functions typically associated with the respective σ factors. Figure 4D presents the top ten gene ontology (GO) terms enriched in genes positioned downstream of predicted promoters across the species shown in Fig. 4C. The σ70/σ38-driven genes primarily relate to fundamental cellular processes, including transcription and translation, along with their regulation, reflecting σ70’s role as the housekeeping σ factor^25^. The σ54-regulated genes show enrichment in functions relating to flagellar motility and nitrogen utilisation, representing known σ54-related functions^26^. Genes downstream of σ32 promoters are strongly associated with protein folding, protein refolding, chaperone functions, and heat response pathways, matching its canonical role in heat shock response^27^. The σ28-regulated genes also demonstrate clear enrichment in known associated roles such as chemotaxis, signal transduction, and flagellum-related functions^28^. Finally, genes controlled by σ24 promoters show associations with envelope stress, osmotic stress, and heat response, consistent with its function in regulating extracytoplasmic stress responses^29^. This consistency between predicted promoter annotations and expected biological functions provides further validation of PromoterAtlas-annotation accuracy in identifying genuine σ factor binding sites.

The precision-optimised configuration employed here identifies promoters conservatively, annotating an average of 15% of extracted regulatory regions across the analysed species (ranging 12–21% depending on the organism, Supplementary Fig. S6). For applications requiring broader coverage prioritising sensitivity over specificity, recall-optimised parameters (1 nucleotide predicted as belonging to promoter class, no co-occurrence enforcement) identify substantially more promoters, spanning an average of 70% of regulatory regions across species (Supplementary Fig. S6).

### Cross-species structure in the PromoterAtlas embedding space

Given PromoterAtlas’s ability to identify promoter motifs belonging to different σ factors in diverse species spanning the Gammaproteobacteria, we sought to examine how the model represents regulatory information across species. To explore this question, we extracted sequence embeddings from various layers of the PromoterAtlas-base model for promoters from multiple organisms and σ factor types. For each sequence, we applied global max pooling across the sequence length to create a fixed-length vector representation that captures the most salient features detected by the model. These sequence-level embeddings were then visualised using Uniform Manifold Approximation and Projection (UMAP)^30^ to reveal clustering patterns across species and σ factor types.

**Fig. 5:**
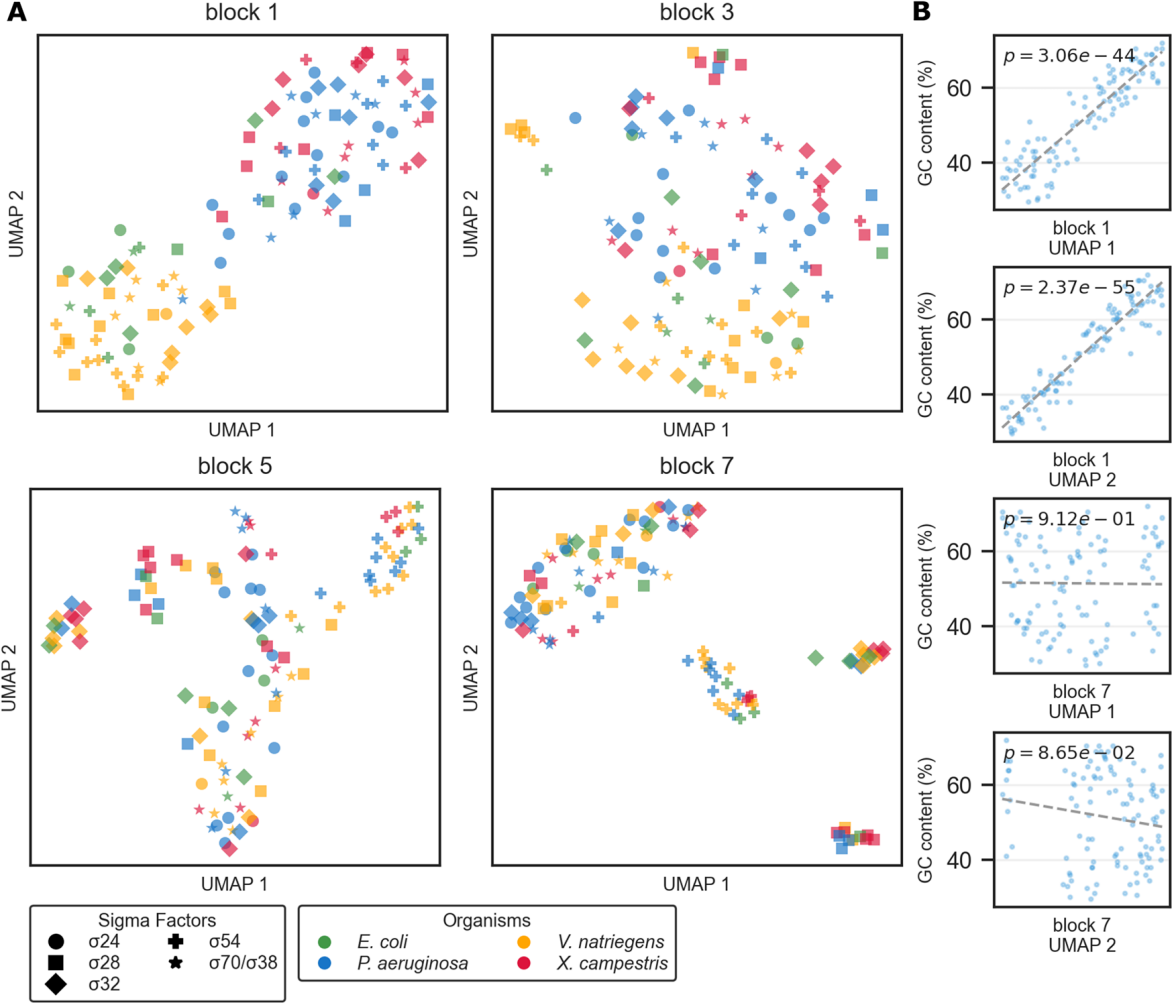
Visualisation of embedding space representations of promoters of various σ type belonging to *E. coli*, *V. natriegens*, *P. aeruginosa*, and *X. campestris* in different PromoterAtlas-base layers. **A** UMAP dimensionality reduction plot of the pooled embeddings of various σ type promoters extracted from the sequence representations following DNATransformer blocks 1, 3, 5, and 7. **B** Scatter plots of sequence GC content against UMAP dimensions 1 and 2 from block 1 and block 7. Correlation was assessed using Pearson’s correlation coefficient (two-sided test; *n* = 121 promoter sequences, df = 119). Block 1: UMAP 1 (*p* = 3.06 × 10^−44^) and UMAP 2 (*p* = 2.37 × 10^−55^) show significant correlation with GC content. Block 7: UMAP 1 (*p* = 9.12 × 10^−1^) and UMAP 2 (*p* = 8.65 × 10^−2^) show no significant correlation.

Analysis of embeddings across different network layers (after DNATransformer blocks 1, 3, 5, and 7) revealed a progressive shift in the model’s representation space. In the shallow layers (particularly after block 1), the primary organising principle appeared to be genomic GC content, with high-GC organisms *P. aeruginosa* and *X. campestris* forming a distinct cluster from a lower-GC cluster with *E. coli* and *V. natriegens*, regardless of promoter type (Fig. 5). However, as information is passed through to deeper layers, σ factor identity emerged as the dominant organising principle. By block 7, promoters from the same σ factor class clustered together across species boundaries, with distinct groupings observed for σ54, σ32, and σ28 promoters. This hierarchical organisation of the PromoterAtlas-base embedding space is consistent with the shared evolutionary conservation of σ factor binding motifs across Gammaproteobacterial species. The transition from GC content to σ factor-based organisation may reflect the evolutionary history of these regulatory elements, where core promoter recognition mechanisms likely predate species-specific GC content biases.

In the visualisation of the block 7 embedding space, some instances of σ54, σ32, and σ28 promoters were clustered with the main heterogeneous central grouping rather than their σ-specific smaller clusters. To investigate this, we looked for the positions in the sequences that most strongly contributed to the pooled sequence embeddings. For promoters within the distinct σ factor clusters, we identified a consistent pattern where specific positions in the embedding space were consistently activated regardless of where the characteristic motifs appeared in the input sequence (shown for σ28 in Supplementary Fig. S7). This suggests that the model employs a positional encoding mechanism to group functionally related promoters. However, whether there is a biological basis for why some promoters of a given σ type are represented through this mechanism while others are not remains unclear.

### Predicting transcription and translation levels

To evaluate PromoterAtlas’s ability to capture functional regulatory information beyond motif identification, we assessed its performance in predicting gene expression at both transcription and translation levels.

**Fig. 6:**
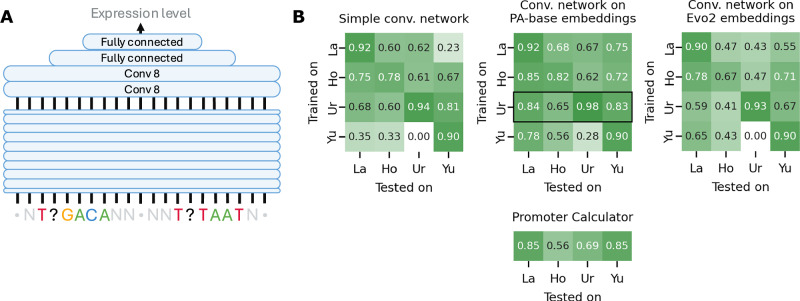
A prediction head leveraging the PromoterAtlas-base embedding enables sequence-based prediction of transcription levels. **A** Architecture of the prediction head: two convolutional layers (kernel width 8) followed by pooling and fully connected layers transform embeddings into predicted expression values. The same architecture was applied to one-hot encoded sequences (4 channels), PromoterAtlas embeddings (128 dimensions), and Evo2^3^ embeddings (4096 dimensions), with convolutional layers maintaining their respective input dimensionalities **B** Heatmaps showing the Pearson correlations between predicted and observed transcription levels for four approaches and tested on four datasets: Lafleur et al.^31^, Hossain et al.^32^, Urtecho et al.^33^, Yu et al.^34^. Each row in every heatmap shows a model trained on one dataset (labelled on left) and evaluated across the test sets of all four datasets (columns). The PromoterAtlas-based model trained on Urtecho et al.^33^ achieves correlations matching or exceeding Promoter Calculator, establishing state-of-the-art sequence-based transcription prediction.

For transcription prediction, we applied an identical convolutional prediction head to three input representations: one-hot encoded sequences (4 channels) as baseline, PromoterAtlas-base final layer embeddings (128 dimensions, Fig. 6A), and Evo2^3^ final layer embeddings (4096 dimensions). Models were trained and evaluated on four independent datasets: Lafleur et al.^31^ (5392 sequences), Hossain et al.^32^ (4350 sequences), Urtecho et al.^33^ (10,898 sequences), and Yu et al.^34^ (1494 sequences). To assess generalisation, we trained four separate models, each on 80% of one dataset with 10% held out for early stopping. Each model was then evaluated on the 10% test splits from all four datasets, with these splits held constant across all evaluations.

The PromoterAtlas-based approach consistently outperformed both the simple convolutional baseline as well as the Evo2 embeddings across all datasets (Fig. 6B, Supplementary Data 1), indicating its final layer embedding representations more task-relevant information for transcription level prediction than the traditional one-hot encoding approach and the Evo2 embeddings. The PromoterAtlas-based model trained on the Urtecho et al.^33^ dataset was the best overall model and achieved Pearson correlations either matching or exceeding Promoter Calculator^31^ across all four datasets, suggesting state-of-the-art performance for sequence-based transcription prediction.

We applied an identical methodology for the prediction of protein expression levels, accounting for both transcription and translation. For this task, we used data from Kosuri et al.^35^, which included combined promoter and RBS sequences with corresponding protein expression measurements. Using the PromoterAtlas-base embeddings with the same regression head design and 80:10:10 split, the trained protein expression model reached a Pearson correlation of 0.79 on the test set (Supplementary Data 1, Supplementary Fig. S8). This approaches the 0.82 reported by Kosuri et al.^35^ using an ANOVA model with explicit RNA-protein interaction terms, demonstrating that PromoterAtlas embeddings capture regulatory information governing both transcription and translation from sequence alone.

## Discussion

In this work, we presented PromoterAtlas, a transformer-based model trained on 9 million regulatory sequences from 3371 gammaproteobacterial species. We demonstrated that PromoterAtlas successfully recognises diverse regulatory elements, including promoters for five different σ factors, RBS, and terminators across the gammaproteobacterial clade. By leveraging the base model embeddings, we developed a genome-wide promoter annotation tool capable of identifying σ factor promoters. Furthermore, we showed that the model’s embedding space encodes meaningful relationships between regulatory elements of different bacteria, and that PromoterAtlas embeddings enable state-of-the-art performance in predicting transcription and translation levels from sequence alone.

A key methodological advance in this work was our approach to data collection. Traditional promoter prediction methods rely on small sets of experimentally validated sequences, typically from single species, which limits both dataset size and generalisability. Conversely, recent genomic foundation models are trained on whole genomes across all domains of life, optimising for broad genomic coverage rather than regulatory sequence specificity. Our approach occupies a middle ground: by systematically extracting sequences upstream of genes belonging to 3371 species within the gammaproteobacterial clade, we created a dataset three orders of magnitude larger than typical supervised approaches whilst maintaining phylogenetic and task-oriented focus. This exploits evolutionary conservation of regulatory architecture within the clade while providing sufficient diversity for the model to learn generalisable patterns through unsupervised pretraining. The unsupervised approach enables the model to discover sequence patterns directly from data without requiring explicit feature engineering, allowing learned representations to capture whatever sequence properties prove predictive for regulatory function. The success of this strategy suggests that taxonomically focused transformer models may provide advantages over both narrow single-species methods and broad general-purpose genomic models for specialised regulatory sequence analysis.

The logit plots introduced in this work represent a distinct approach to examining the features of gammaproteobacterial regulatory sequences, enabling the identification of promoters, ribosomal binding sites, transcription factor binding sites, terminators, and stop codons. This approach offers an initial visual screening method for researchers examining regulatory sequences, with attention maps providing finer resolution when broad regions of elevated logit values prevent easy identification of individual motifs.

Our work presents an end-to-end whole-genome annotation tool specifically designed for bacterial promoters across a family of bacterial species, addressing a gap in existing computational workflows. Beyond performance metrics, this addresses critical workflow limitations in existing tools. While several promoter prediction tools exist in the literature, many rely on web interfaces limited to single-sequence analysis. The annotation pipeline we developed enables direct processing of GenBank, GFF3, and FASTA files, facilitating rapid characterisation of newly sequenced bacterial genomes. We validated this approach through multiple strategies: comparison to *E. coli* ChIP-seq data and RegulonDB, analysis of predicted σ-type promoter distributions, and gene ontology enrichment studies. The annotation method parameters are adjustable, allowing users to tune precision-recall trade-offs based on their specific applications.

Current expression prediction tools for bacterial systems are primarily developed for and validated in *E. coli* and other model organisms, creating a need for more broadly applicable approaches. While the expression prediction models presented in this work were also trained and validated using *E. coli* datasets due to gene expression data availability constraints, PromoterAtlas’s underlying base model was trained across the entire gammaproteobacterial clade. This broader phylogenetic training foundation suggests that our approach may be more readily extensible to expression prediction in non-model bacteria compared to existing tools. This hypothesis is supported by our segmentation model results and UMAP analysis, which demonstrate that the later-layer embeddings encode regulatory information that transcends species boundaries. Nevertheless, comprehensive cross-species validation of expression prediction capabilities remains challenging due to the scarcity of standardised expression datasets for non-model organisms.

PromoterAtlas also achieved competitive expression prediction performance to PromoterCalculator and outperformed the foundation model Evo2 using final-layer embeddings and a convolutional regression head. This performance difference may reflect task-specific training: PromoterAtlas was pretrained solely on regulatory sequences, whereas Evo2 was optimised for next-token prediction across whole genomes spanning the entire tree of life. Beyond performance, the difference in model scale (PromoterAtlas: 1.8 M parameter backbone vs Evo2: 7B) has practical implications. Lightweight models trained on specific genomic feature classes can be deployed on standard laboratory hardware, whereas foundation models require substantial GPU resources. Focused pretraining may therefore offer advantages for specialised tasks where whole-genome context is not required.

Looking forward, PromoterAtlas opens several avenues for future research. The model’s ability to identify diverse σ factor binding sites creates opportunities for strength prediction of promoters across different σ factors, potentially extending to transcription factor-dependent expression prediction. Such tools could enhance the understanding of bacterial gene regulation beyond the standard housekeeping promoters. In synthetic biology, accurate prediction and design of promoter strength across different regulatory contexts could facilitate more precise engineering of bacterial gene expression systems. For basic research, the model offers tools for identifying adaptation strategies through regulatory analysis and suggesting potential biological functions for uncharacterised proteins based on their upstream regulatory elements. The embedding space of PromoterAtlas may also enable novel approaches to cluster analysis for identifying co-regulated genes across diverse bacterial species.

## Methods

### Dataset generation

The dataset used for base model training in this study was created by using the NCBI API to query the NCBI genomes database^36^ for genomes of Gammaproteobacteria with annotated coding sequences. Only bacteria with unique species names were retained to prevent identical or highly similar DNA sequences occurring in the dataset. Sequences spanning 200 nucleotides upstream of any annotated coding sequence were extracted and added to the dataset, with the exception of coding sequences occurring within less than 50 nucleotides from the previous coding sequence to avoid including coding sequences belonging to the same operon and therefore potentially lacking promoter or other regulatory features. The length of 200 was chosen to enrich the training data with proximal regulatory elements, as more distal regulatory elements are rare in bacteria, and longer sequences would reduce the overall density of regulatory features in the data. Standardising the length for each sequence additionally enhanced computational efficiency during training by allowing large batches of shuffled same-length sequences. For sequences on the negative strand, the reverse complement was extracted such that all sequences represent the 5′ to 3′ coding strand orientation.

### PromoterAtlas-base architecture

Sequences are one-hot encoded before passing to the PromoterAtlas-base network. The network itself starts with an encoder consisting of a linear projection from the one-hot-encoded dimension of four (A, C, G, T) to a hidden dimension of 128. The resulting representation is subsequently transformed by eight DNATransformer blocks, before being projected back to the dimension four by a linear decoder to enable comparison to the missing nucleotide tokens for loss calculation (Fig. 1A). A DNATransformer block consists of three elements, which maintain the sequence length of 200 and each have residual connections. The first element is a double convolution with window size 3. The second element is a rotary attention layer, which is a variation of classical attention where positional information is encoded by rotating the query and key token vectors of the attention operator, in a frequency-dependent manner where different rotation frequencies are applied to different vector dimensions^37^. This allows the attention mechanism to encode relative positions between features in the sequence, which is well suited to the nature of DNA regulatory sequences, where the distance between motifs is highly relevant to their function. The third element of the DNATransformer block is a feed forward layer, consisting of a linear projection from dimension 128 to dimension 256 and subsequently a linear projection back to dimension 128.

### PromoterAtlas-base training

PromoterAtlas-base training was performed by zero-masking 20 positions randomly selected in every sequence, and making the model predict the missing nucleotides. This encourages the model to focus on patterns in the data that can help to determine what nucleotides were masked. The dataset was split into a training set and validation set in a 9:1 ratio. Categorical cross-entropy loss was applied to the masked positions only as the training criterion, using the AdamW optimiser. A batch size of 1024 and initial learning rate of 10^−3^ were used. The learning rate was scheduled to be reduced by half with a validation loss patience of 5 epochs. Early stopping was scheduled with a patience of 15 on the validation set. PromoterAtlas-base was trained on a single NVIDIA a100, and took 264 epochs (~5 days) to converge. A training loss plot can be found in Supplementary Fig. S1.

### PromoterAtlas-base logit plots

The PromoterAtlas-base logit plots are created by extracting the pre-softmax output logits of an input sequence. For each position in a sequence, the value corresponding to the sequence’s nucleotide in that position is used to plot the height of that nucleotide’s letter in the plot. For the attention map plots, the attention matrices for a sequence are extracted from each of the model’s rotary attention modules and subsequently averaged per position. The per-position per-nucleotide averages are subsequently plotted in analogy to the logit plots.

### PromoterAtlas-annotation

To develop an automated genome-wide promoter annotation tool, we trained a lightweight segmentation head on top of the pretrained PromoterAtlas-base model using a transfer learning approach (Fig. 4A). The training data consisted of 318 regulatory sequences spanning 200 nucleotides upstream of coding sequences, in which we manually annotated promoter motifs that were clearly identifiable in the sequences’ logit plots and attention maps. This resulted in a dataset containing 147 σ70/σ38 promoters, 50 σ54 promoters, 76 σ32 promoters, 27 σ28 promoters, and 18 σ24 promoters. The σ70 and σ38 promoter motifs were grouped together for the segmentation task as they exhibit highly similar consensus sequences that preclude visual discrimination, given their frequent overlapping affinity for both σ factor polymerase complexes^38^. These sequences were selected from diverse gammaproteobacterial species (excluding *E. coli* to enable independent validation) by examining upstream regions where PromoterAtlas-base logit plots exhibited clear, unambiguous promoter patterns. To ensure representation across different sigma factors, we prioritised examination of genes from functional categories typically regulated by each sigma factor, then selected those sequences where logit plots showed the clearest motif patterns matching known consensus sequences. The precise locations of promoter elements (−10 boxes, −35 boxes, etc.) were manually annotated based on peak positions in logit plots and attention maps, retaining only sequences with the most visually obvious promoter motif occurrences.

The segmentation model architecture consisted of the pretrained PromoterAtlas-base backbone with frozen weights, followed by a shallow trainable segmentation head. The feature embeddings from the final layer of the backbone were fed into the segmentation head, which consisted of three sequential 1D convolutional layers (kernel size 10, stride 1, with “same” padding). The final convolutional layer projected the features to a 12-dimensional output space, corresponding to the number of possible sequence annotations at each position (e.g., “no feature”, “σ70/σ38 -10 promoter motif”, “σ70/σ38 −35 promoter motif”, etc.). Training was performed using cross-entropy loss and the Adam optimiser with a learning rate of 10^−5^ and a batch size of 5. Model performance was monitored on a validation set (20% of the data), with early stopping implemented using a patience of 10 epochs on validation loss.

For end-to-end whole-genome annotation of GenBank files, all 200 nucleotide sequences upstream of coding sequences were automatically extracted. Subsequently, promoter features were predicted using the PromoterAtlas-annotation model. To optimise annotation precision, we performed sensitivity analysis on segmentation parameters, varying the threshold for how many consecutive nucleotides with the same prediction are required to constitute a valid regulatory segment (1–6 positions) and toggling co-occurrence enforcement of paired promoter elements (−10/−35 boxes). Co-occurrence rules enforce biological consistency by ensuring paired promoter elements (e.g., −10/−35 motifs) appear together for each sigma factor (σ70/σ38, σ54, σ32, σ28, and σ24). Precision and recall were evaluated by validating predictions against the Cho et al.^21^ ChIP-seq dataset for *E. coli*. Maximum precision was achieved using a threshold of 3 consecutive positions with co-occurrence enforcement enabled, while maximum recall was achieved using 1 consecutive position without co-occurrence enforcement. The high-precision parameters (3 consecutive positions with co-occurrence enforcement) were used for conservative annotation of representative gammaproteobacterial species presented in the main figures. The high-recall parameters (1 consecutive position without co-occurrence enforcement) were used for the analysis in Supplementary Fig. S6. Following segmentation, predicted regulatory features were mapped back to their genomic coordinates with strand-specific adjustments and integrated into the original GenBank files as annotated regulatory elements with appropriate feature qualifiers. This automated pipeline facilitated large-scale annotation of bacterial genomes, enabling systematic identification of promoter features across multiple species.

Predicted promoters were validated against two independent benchmarks. For ChIP-seq validation, promoter predictions were compared to σ factor binding regions from Cho et al.^21^. A prediction was considered validated if either the −10 or −35 box coordinates overlapped with a ChIP-seq binding region for the corresponding σ factor. For RegulonDB validation, the full promoter sequence spanning from the −35 to −10 box (reverse complemented for genes on the negative strand) was extracted and compared against RegulonDB promoter sequences. A prediction was validated if the extracted sequence matched or was contained within any RegulonDB entry for that σ factor.

### Gene ontology analysis

To functionally characterise genes regulated by different σ factors, we performed Gene Ontology (GO) term enrichment analysis on genes downstream of predicted promoter motifs. For each annotated promoter identified by PromoterAtlas-annotation, we extracted the locus tag or gene name of the downstream coding sequence from the GenBank files. We then queried the UniProt REST API (https://rest.uniprot.org) to retrieve the associated GO terms for each gene. For each σ factor category, we compiled the frequency of associated GO terms and identified the top ten most enriched terms.

### Embedding space analysis

We selected a subset of regulatory sequences classified as promoters belonging to the different σ factors by PromoterAtlas-annotation from *E. coli, V. natriegens, P. aeruginosa*, and *X. campestris*. For each of the sequences, we extracted the embedding from multiple layers of the network (blocks 1, 3, 5, and 7), applying global max pooling across the sequence dimension to create fixed-length vector representations (length equal to the hidden dimension 128 of PromoterAtlas-base). The resulting sequence embeddings were then visualised using Uniform Manifold Approximation and Projection (UMAP)^30^ to reduce dimensionality while preserving local and global structure. UMAP parameters were set to n_neighbors=15, min_dist=0.1, and n_components=2. Before UMAP transformation, embeddings were standardised to ensure equal weighting of each dimension. For Supplementary Fig. S5, we identified the positions that yielded the maximum value for each dimension in the embedding space and tallied their frequency. These position counts were then visualised alongside logit plots to reveal potential relationships between sequence features and embedding patterns, using colour intensity to represent the number of dimensions along which each position contributed the maximum value for the global max pooling.

### Prediction of transcription levels

We obtained the datasets for the prediction task of transcription levels of promoter sequences from various sources. From Lafleur et al.^31^, we obtained 5392 promoter sequences, from Hossain et al.^32^, we obtained 4350 promoter sequences, from Urtecho et al.^33^, we obtained 10,898 promoter sequences, and from Yu et al.^34^, we obtained 1494 sequences. All sequences were standardised to 86 nucleotides, corresponding to the longest sequence across all four datasets. Shorter sequences were padded with adenine nucleotides.

The simple convolutional baseline operated directly on one-hot encoded DNA sequences (4 input channels). The PromoterAtlas-based model used last layer embeddings from the pre-trained PromoterAtlas-base backbone (128-dimensional) with frozen weights. The Evo2^3^-based model used embeddings from the evo2_7b model (7 billion parameters), extracted from the final layer (blocks.31), yielding 4096-dimensional representations for each nucleotide position. The regression head architecture was based on the architecture for expression prediction from embeddings in the original Evo paper^39^. All three models used identical prediction head architectures consisting of two 1D convolutional layers (kernel size 8, stride 1, “same” padding), max pooling (kernel size 7, stride 1, padding 3), and two fully connected layers with ReLU activations between layers (dimensions: flattened→64 → 1). The convolutional layers operated at the following hidden dimensions: the baseline model projected from 4 to 128 dimensions in the first convolutional layer and maintained 128 dimensions in the second convolution thereafter; the PromoterAtlas-based model maintained 128 dimensions throughout; and the Evo2-based model maintained 4096 dimensions throughout. Due to the computational cost of generating Evo2 embeddings, these were pre-computed for all sequences across all datasets and cached prior to training. Due to high activations, the Evo2 embeddings were normalised using layer normalisation prior to being fed into the regression head.

Training for both models was performed using the AdamW optimiser with a learning rate of 10^−5^ and batch size of 32. Mean squared error was used as the loss function, with model performance monitored on a validation set (10% of data). Early stopping was implemented with a patience of 10 epochs on validation loss, and the remaining 10% of data was reserved for testing. Target transcription values were z-score normalised using the mean and standard deviation computed exclusively from each dataset’s training set (80% of the data). These normalisation parameters were also applied to the corresponding validation and test sets from the same dataset during evaluations. For cross-evaluation, separate models were trained on each training set and evaluated against all other test sets to assess generalisation capabilities. Performance was measured using Pearson correlation between predicted and observed transcription levels, with both model predictions and PromoterCalculator^31^ predictions compared on the same test sets.

For Promoter Calculator predictions^31^, we retained the minimum predicted dG_total across the forward sequence as the prediction score.

### Prediction of protein expression levels

For the protein expression level prediction task accounting for both transcription and translation, we used data from Kosuri et al.^35^, which included combined promoter and RBS sequences with corresponding protein expression measurements. Following our approach for transcription prediction, we standardised all sequences to a maximum length of 86 nucleotides, with shorter sequences padded with adenine nucleotides. For expression level prediction, we employed the same architectural design as our transcription prediction model, based on the pre-trained PromoterAtlas-base backbone with frozen weights. As with the transcription model, feature embeddings from the backbone were processed through a custom prediction head consisting of two 1D convolutional layers (kernel size 8, stride 1, with “same” padding), max pooling (kernel size 7, stride 1, padding 3), and two fully connected layers (dimensions 128 × 86 → 64 → 1) with ReLU activations.

Given the wide dynamic range of protein expression values in the dataset, we applied a logarithmic transformation (log(count+1)) to the target values before normalising. The training procedure followed the same approach as our transcription prediction models, using the AdamW optimiser, with learning rate of 10^−5^ and early stopping patience of 10 epochs. We maintained the same train/validation/test split ratio (80%/10%/10%) and evaluated model performance using Pearson correlation between predicted and observed protein levels after reversing the normalisation and logarithmic transformations.

### Reporting summary

Further information on research design is available in the Nature Portfolio Reporting Summary linked to this article.

## Supplementary information

Supplementary Information

Description of Additonal Supplementary Files

Supplementary Data 1

Reporting Summary

Transparent Peer Review file

## Data Availability

The data used in this study to train the base model as well as the manually annotated promoter motif data for the segmentation model are available at https://huggingface.co/datasets/LCoppens/PromoterAtlas-data.

## Code availability

The PromoterAtlas software package is available at https://github.com/LucasCoppens/PromoterAtlas. This repository includes model weights, a command-line tool for annotation (supporting GenBank, GFF3 and FASTA formats), scripts for generating logit plot visualisations, and inference scripts for expression prediction. Example annotated genomes for medically and biotechnologically relevant gammaproteobacteria are included. Code to reproduce all analyses and figures in this manuscript is available at https://github.com/LucasCoppens/PromoterAtlas_paper. The versions of both repositories used in this study are archived on Zenodo (PromoterAtlas: 10.5281/zenodo.19223931)^40^ (PromoterAtlas_paper 10.5281/zenodo.19223939)^41^.
