# Supplementary material for: PromoterAtlas: decoding regulatory sequences across Gammaproteobacteria using a transformer model
Source: Nat Commun. 2026 May 15;17:6451. doi: 10.1038/s41467-026-72837-3 (PMC13377108; doi:10.1038/s41467-026-72837-3)
Supplement: Supplementary file 1 — Supplementary Information [file 41467_2026_72837_MOESM1_ESM.pdf]

Supplementary Information

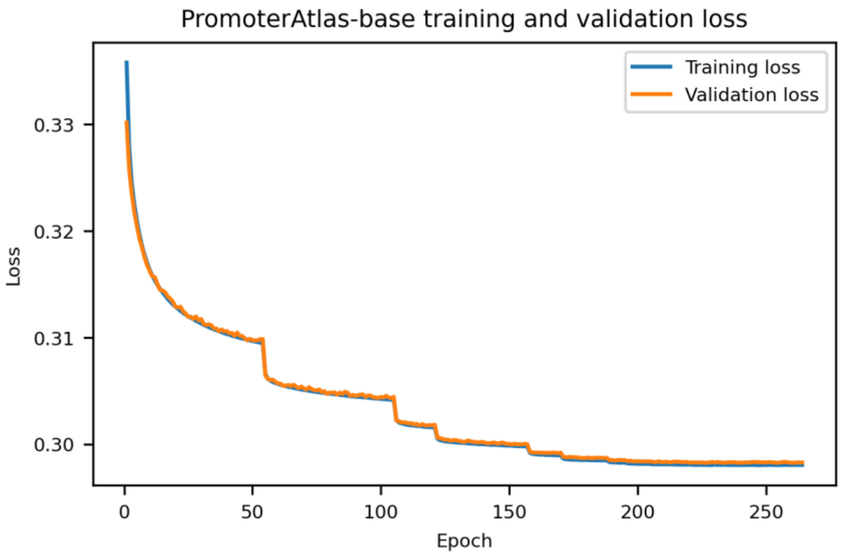

Figure S1. PromoterAtlas-base training loss plot. The curve shows good convergence of train and validation loss. The step-wise behaviour is explained by the learning rate being halved when a plateau is reached with the previous learning rate, allowing the model to learn finer detail each time.

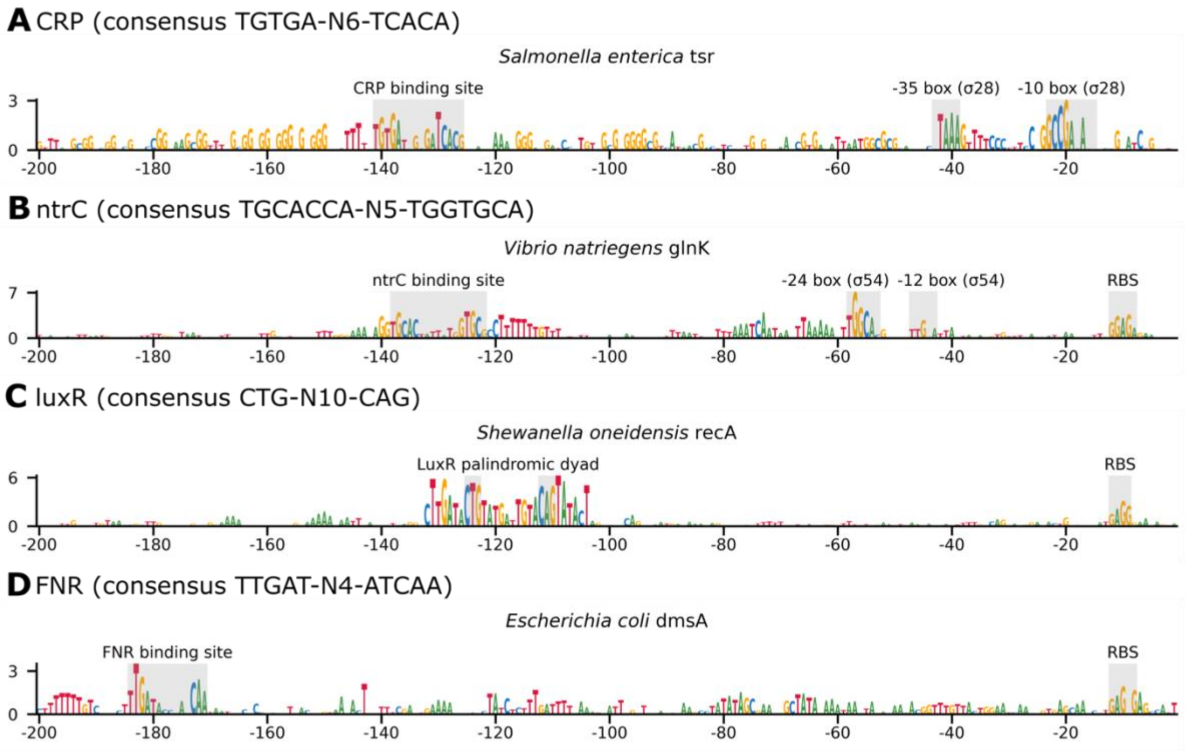

Figure S2. Examples of PromoterAtlas logit plots showing recognition of transcription factor binding site motifs. (A) Logit plot of the regulatory region upstream of *S. enterica* gene tsr shows recognition of a CRP binding site. (B) Logit plot of the regulatory region upstream of *V. natriegens* gene glnK shows recognition of a ntrC binding site. (C) Logit plot of the regulatory region upstream of *S. oneidensis* gene recA shows recognition of a luxR binding site. (D) Logit plot of the regulatory region upstream of *E. coli* gene dmsA shows recognition of a FNR binding site.

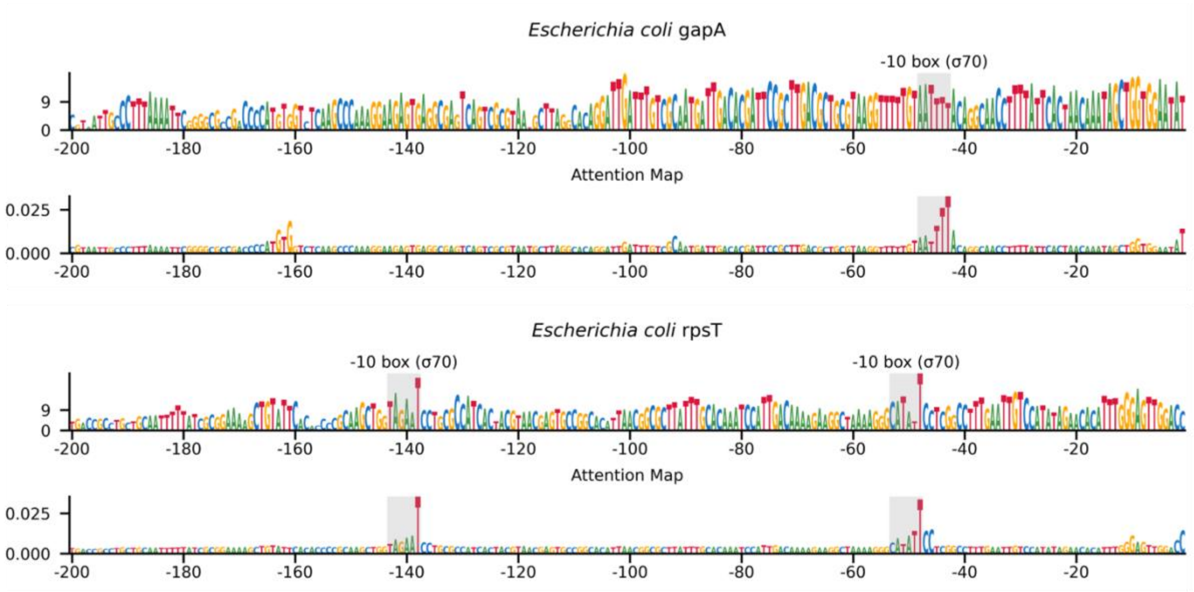

Figure S3. Promoter identification in logit plots with the help of attention maps. In the regulatory regions upstream of some genes known to be highly expressed, long stretches of high logits in the plot prevent straightforward identification of regulatory elements. In those cases, we often found that plotting the PromoterAtlas's average attention map would reveal the presence of σ70 motifs, exhibiting a peak in the logit values at the last position of the -10 motif box.

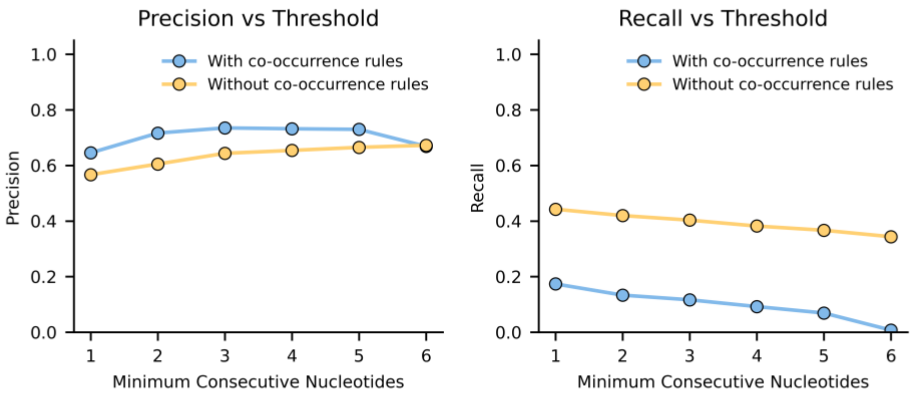

Figure S4. Threshold sensitivity analysis for PromoterAtlas genome annotation. Precision and recall for sigma factor promoter predictions on *E. coli* K-12 (NC_000913.1) evaluated against Cho et al. (2014)[1] ChIP-seq data across different minimum consecutive nucleotide thresholds (1-6). Blue lines show results with co-occurrence rules requiring both -35/-10 (or -24/-12 for σ54) elements to be present; yellow lines show results without co-occurrence filtering. Threshold=3 and co-occurrence rules enabled provides the optimal parameters for high precision annotation, while threshold=1 without co-occurrence enforcement provides optimal configuration for high recall.

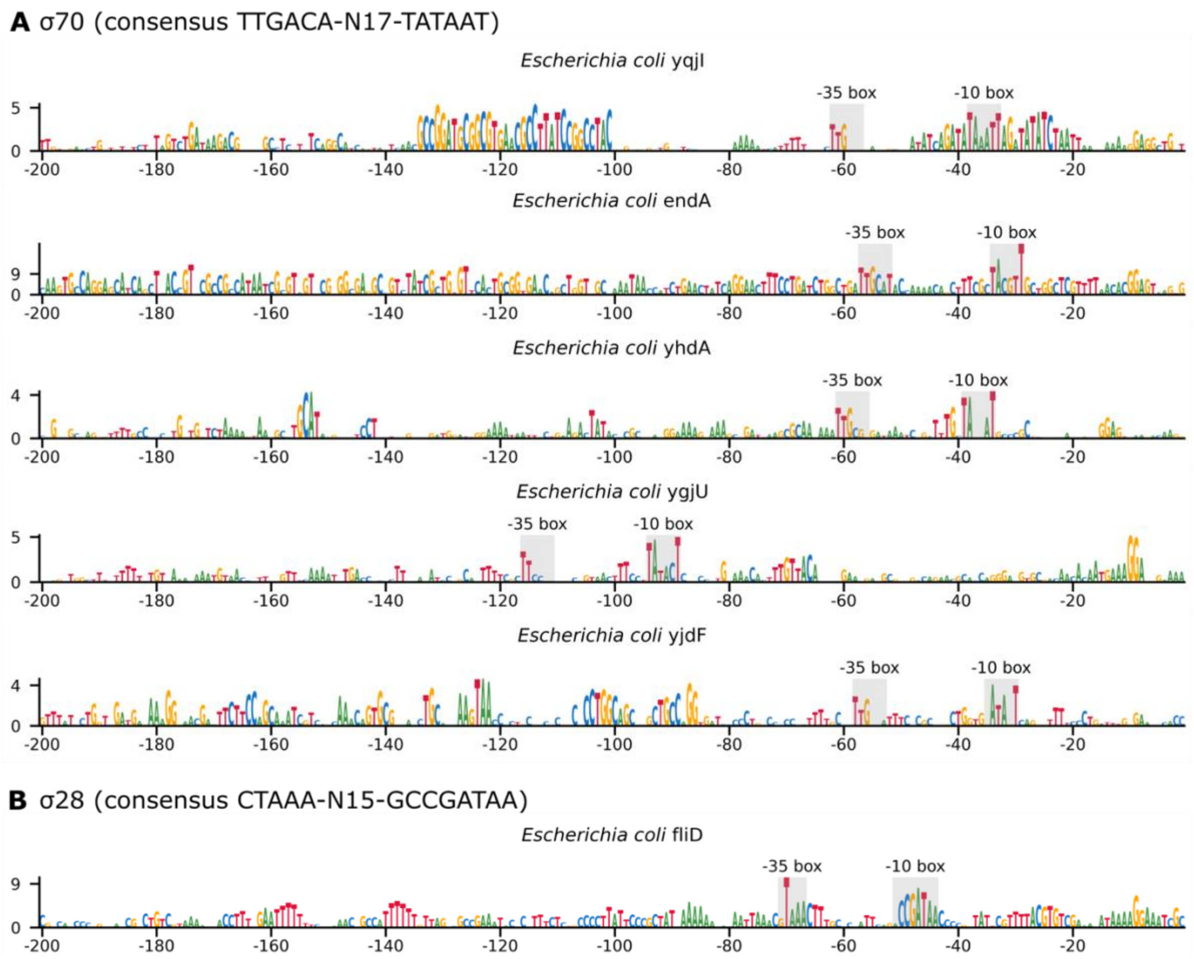

Figure S5. Logit plots of *E. coli* promoters predicted by PromoterAtlas-segmentation but were not validated by either Cho et al. (2014)[1] ChIP-seq data or RegulonDB[2,3] show clear promoter motifs. (A) Logit plots of 5 σ70 promoters predicted by PromoterAtlas-segmentation but not validated by Cho et al. (2014)[1] or RegulonDB. (B) Logit plots of the 1 σ28 promoter predicted by PromoterAtlas-segmentation but not validated by Cho et al. (2014)[1] or RegulonDB.

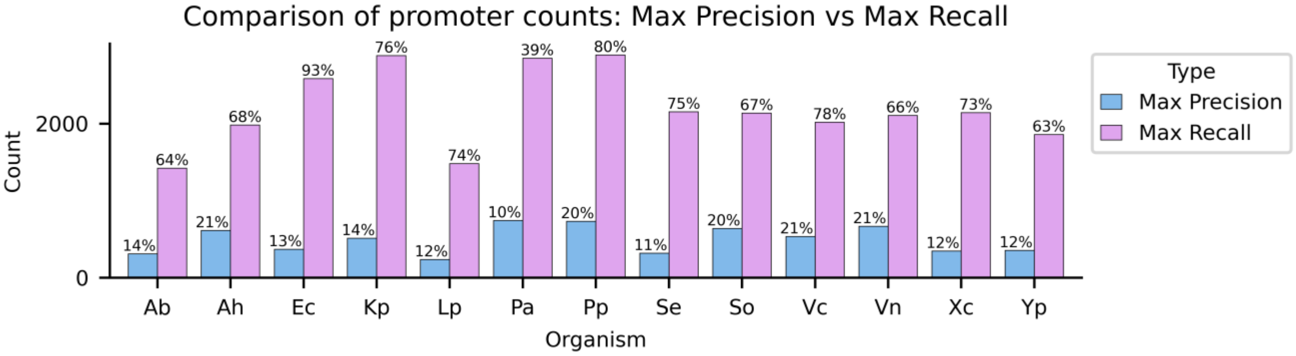

Figure S6. Comparison of precision-optimised versus recall-optimised promoter annotation parameters across gammaproteobacterial species. Bar plot showing the number of promoters identified using precision-optimised parameters (3 consecutive nucleotides with co-occurrence enforcement, blue) versus recall-optimised parameters (1 consecutive nucleotide without co-occurrence enforcement, purple) for 13 species. Percentages indicate the fraction of extracted 200-nucleotide upstream regulatory regions containing at least one annotated promoter. Precision-optimised settings annotate 10-21% of regions with high confidence, while recall-optimised settings achieve 63-93% coverage with reduced stringency. Organism abbreviations as in Figure 4

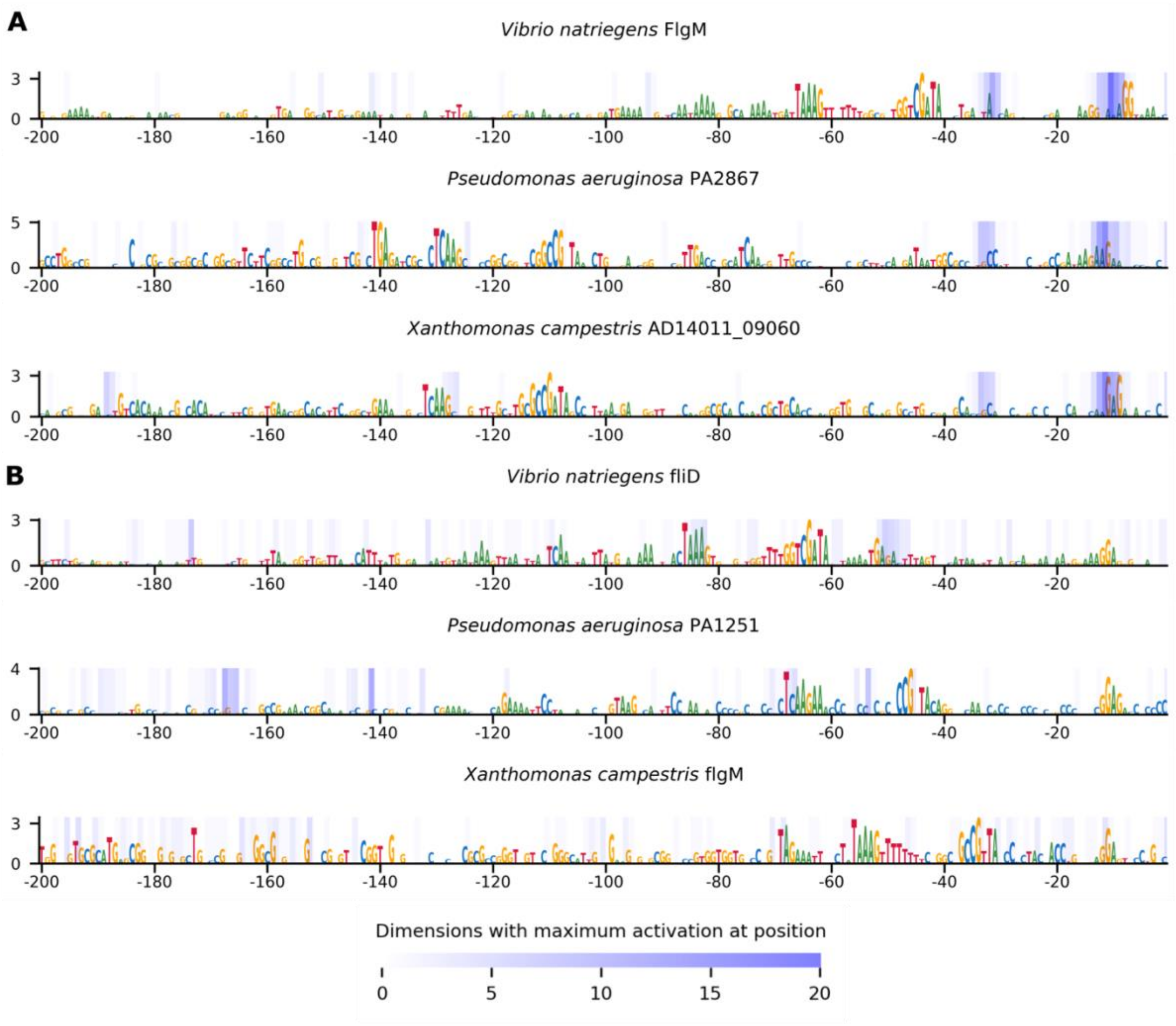

Figure S7. Visualisation of positions contributing most strongly to pooled sequence embeddings reveal two distinct encoding mechanisms for σ28 promoter sequences. (A) The embeddings of three σ28 promoter sequences from the σ28-exclusive cluster in the block 7 UMAP plot from 5are mostly determined by information encoded in the vicinities of the -10 and -33 positions, regardless of where the σ28 promoter motifs appear in the sequence. (B) Three examples of σ28 promoter sequences from the main heterogeneous cluster in the block 7 UMAP plot from Figure 5 which do not show the same characteristic encoding pattern as the σ28-specific cluster.

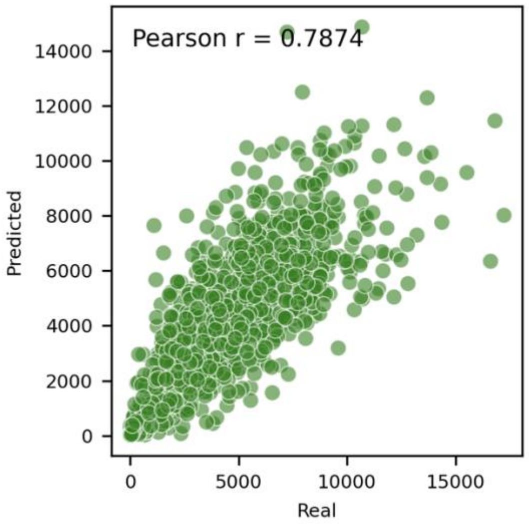

Figure S8. Scatter plot of real protein expression values from the Kosuri et al. (2013)[4] dataset and the values predicted by the PromoterAtlas protein expression model on the test data.

Supplementary References

1. Cho, B. K., Kim, D., Knight, E. M., Zengler, K. & Palsson, B. O. Genome-scale reconstruction of the sigma factor network in Escherichia coli: Topology and functional states. *BMC Biol.* **12**, 1–11 (2014).
2. Salgado, H. *et al.* RegulonDB v12.0: a comprehensive resource of transcriptional regulation in E. coli K-12. *Nucleic Acids Res.* https://doi.org/10.1093/nar/gkad1072 (2023) doi:10.1093/nar/gkad1072.
3. Brixi, G. *et al.* Genome modeling and design across all domains of life with Evo 2. *bioRxiv* 2025.02.18.638918 (2025).
4. Kosuri, S. *et al.* Composability of regulatory sequences controlling transcription and translation in Escherichia coli. *Proc. Natl. Acad. Sci. U. S. A.* **110**, 14024–14029 (2013).
