# Supplementary material for: PromoterAtlas: decoding regulatory sequences across Gammaproteobacteria using a transformer model
Source: Nat Commun. 2026 May 15;17:6451. doi: 10.1038/s41467-026-72837-3 (PMC13377108; doi:10.1038/s41467-026-72837-3)
Supplement: Supplementary file 2 — Description of Additonal Supplementary Files [file 41467_2026_72837_MOESM2_ESM.pdf]

Description of Additional Supplementary Files

Supplementary Data 1

Expression prediction performance metrics. Each row reports the performance of a given model for a given training dataset and evaluation dataset
