# Supplementary material for: PromoterAtlas: decoding regulatory sequences across Gammaproteobacteria using a transformer model
Source: Nat Commun. 2026 May 15;17:6451. doi: 10.1038/s41467-026-72837-3 (PMC13377108; doi:10.1038/s41467-026-72837-3)
Supplement: Supplementary file 5 — Transparent Peer Review file [file 41467_2026_72837_MOESM5_ESM.pdf]

nature portfolio

Peer Review File

PromoterAtlas: decoding regulatory sequences across gammaproteobacteria using a transformer model

Corresponding Author: Professor Rodrigo Ledesma-Amaro

This file contains all reviewer reports in order by version, followed by all author rebuttals in order by version.

Version 0:

Reviewer comments:

Reviewer #1

(Remarks to the Author)
In the article "PromoterAtlas: decoding regulatory sequences across Gammaproteobacteria using a transformer model," the authors describe a method to address the challenge of mining highly diverse promoters and regulatory DNA elements across a broad range of bacteria and classifying them.
The power of transformer models for learning protein or DNA sequence patterns has been demonstrated in many recent studies. The architecture of the PromoterAtlas-base model appears to be very effective.
Major
Introduction
- In the introduction, the authors describe that deep learning approaches hardly perform better than HMM- or PWM-based prediction methods. However, many studies have shown that machine learning approaches such as CNN or CNN-LSTM models outperform these traditional methods.
- Indeed, several publications focus on Escherichia coli, but many other species have also been subjected to promoter prediction with successful results published.
Results
Recognition of regulatory features
minor
- The authors choose to use 200 nt upstream regions, why no just the intergenic region?
Whole genome promoter motif annotation
Major
"we manually annotated based on logit plots, consisting of 147 σ70/σ38 promoters, 50 σ54 promoters, 76 σ32 promoters, 27 σ28 promoters, and 18 σ24 promoters." It is unclear on what basis these promoters were selected. Why is this set considered better than known sequences? Moreover, it seems very limited that only 300–400 sequences out of 9 million were chosen to train the model. If these were selected based on knowledge derived from E. coli, this would be a poor choice since E. coli is used as the "leave one out" organism for validation.
This is a strange assumption: "56% for a naive baseline scenario in which σ70 promoters are systematically placed 30 nucleotides upstream of every coding sequence." This is simply reflecting biological organization — promoter regions are typically located upstream of coding sequences, and most of them are σ70 promoters. The real challenge lies in accurately predicting the Transcription Start Site (TSS) and its associated promoter elements.
"the smallest amount of promoters predicted in Legionella pneumophila (206), and the largest amount of promoters detected in P. aeruginosa (615)." These numbers are surprisingly low, as several thousand promoters would be expected. P. aeruginosa has 3,932 regions, meaning that a promoter was found in only about 15% of them. Please provide the relevant metrics to clarify this result.
Existing programs such as STREME (from the MEME suite) can also identify the same promoter patterns shown here. Does the PromoterAtlas-base model outperform STREME?
Predicting transcription and translation levels
Looks very promising, with a Pearson correlation of 0.79 on the test set. However, it was somewhat difficult to verify this in detail based on the results provided. This analysis could potentially form an article on its own and may be somewhat beyond the scope of the current manuscript.Discussion & perspectives
Major
"capture subtle sequence features that influence DNA physical properties such as melting temperature or topological characteristics. These features are typically overlooked by traditional position weight matrices or other methods exclusively

focussed on motifs.” This is indeed a limitation of the older PWM-based methods, but such features have been widely incorporated and described in studies employing ML, LLM, or AI models.
“Current expression prediction tools for bacterial systems are primarily developed for and validated in E. coli” and other model organisms.
Software
minor
- The software was easy to install and ran smoothly. The results are provided only in GenBank format; adding support for additional formats would be useful to enable import into other visualization or analysis packages.

(Remarks on code availability)
The software tool is easy to install, is working good, and is user friendly. The output is too limited and additional formats should be added.

Reviewer #2

(Remarks to the Author)
In this manuscript, the authors presented PromoterAtlas, a transformer-based model trained on 9 million regulatory sequences for the identification and annotation of bacterial promoters. The authors demonstrate the model's utility in multiple tasks, including the visualization of regulatory features, genome-wide annotation of promoters for five σ factors, prediction of gene expression levels, and capturing evolutionary relationships in its embedding space. The scale of the pre-training data and the integration of these diverse capabilities into a single, end-to-end framework is a significant strength. The work addresses a important challenge in bacterial genomics. The work is technically sound and describes a significant methodological advancement in the field of computational genomics. However, some major points need to be addressed.
1.The study presents several intriguing observations, such as the encoding mechanisms for σ28 promoters and the phylogenetic relationships captured in the embedding space. However, the current analysis primarily describes these patterns without sufficiently investigating their underlying biological causes or functional consequences. To strengthen the study and move beyond correlation towards causation, it is recommended that the authors attempt to validate a key prediction by biological experiments.
2.The manuscript currently states that sequences upstream of coding regions were extracted but lacks critical methodological details. Specifically, the procedure for handling the directional nature of DNA strands (e.g., how upstream regions were defined and extracted for DNA sequences containing both positive and negative strands) is not explained. A more comprehensive description of the data processing pipeline is essential to ensure the reproducibility of the study.
3.The PromoterAtlas-base model was pre-trained on 200-nucleotide sequences, yet the transcription and translation prediction models were trained and evaluated on sequences standardized to a length of only 86 nucleotides. The authors do not explain the rationale for this significant discrepancy in input length. A detailed justification for this specific length choice and a discussion of its potential impact on model performance and biological relevance are necessary.
4. The threshold for the "minimum of 4 consecutive nucleotides" in the genome annotation pipeline seems arbitrary. Was this parameter optimized? Please provide a justification or a sensitivity analysis.
5.The manuscript suffers from substantial and inappropriate textual overlap between the Methods and Results sections. For instance, the detailed description of the sequence standardization process and the architecture of the prediction models in the "Prediction of transcription levels" (Methods) is virtually identical to the text in the "Predicting transcription and translation levels" (Results). This extensive repetition, from "All sequences were standardised to a maximum length of 86 nucleotides..." to "...but operated on raw sequence one-hot encodings rather than learned embeddings," undermines the manuscript's professionalism. The Results section should focus on presenting and interpreting the findings (e.g., performance metrics, correlation values), not on reiterating methodological details. The Methods section exists to provide the comprehensive technical description required for reproducibility. I strongly recommend that the authors thoroughly revise the entire manuscript to eliminate these redundancies. The Results section should present a clear narrative of the discoveries, while the Methods section should be preserved as the single, authoritative source for the experimental and computational procedures.
6.The authors should more clearly articulate the specific advantages of their PromoterAtlas approach when compared to contemporary general-purpose genomic foundation models, such as Evo and Evo2. The manuscript currently lacks a direct performance comparison with these state-of-the-art models on relevant tasks (e.g., promoter annotation or expression prediction). Given that these models are trained on trillions of nucleotides across diverse species and have demonstrated powerful capabilities in functional element prediction, it is essential to benchmark PromoterAtlas against them. he authors should discuss their method's comparative performance and justify its value.

(Remarks on code availability)

Version 2:

Reviewer comments:

Reviewer #1

(Remarks to the Author)

After carefully reviewing the revised manuscript and the authors’ responses, I am satisfied that all requested modifications and clarifications have been adequately addressed. The manuscript has improved in clarity and quality as a result of these revisions. I therefore recommend acceptance of the manuscript in its current form.

(Remarks on code availability)
The provided scripts are usable and contain all support information

Reviewer #2

(Remarks to the Author)
This paper can be accepted since the authors have revised it according to the comments of the reviewers.

(Remarks on code availability)

Open Access This Peer Review File is licensed under a Creative Commons Attribution 4.0 International License, which permits use, sharing, adaptation, distribution and reproduction in any medium or format, as long as you give appropriate credit to the original author(s) and the source, provide a link to the Creative Commons license, and indicate if changes were made.
In cases where reviewers are anonymous, credit should be given to 'Anonymous Referee' and the source.
The images or other third party material in this Peer Review File are included in the article’s Creative Commons license, unless indicated otherwise in a credit line to the material. If material is not included in the article’s Creative Commons license and your intended use is not permitted by statutory regulation or exceeds the permitted use, you will need to obtain permission directly from the copyright holder.
To view a copy of this license, visit https://creativecommons.org/licenses/by/4.0/

Reviewer #1 (Remarks to the Author):

In the article "PromoterAtlas: decoding regulatory sequences across Gammaproteobacteria using a transformer model," the authors describe a method to address the challenge of mining highly diverse promoters and regulatory DNA elements across a broad range of bacteria and classifying them.

The power of transformer models for learning protein or DNA sequence patterns has been demonstrated in many recent studies. The architecture of the PromoterAtlas-base model appears to be very effective.

Major
Introduction

- In the introduction, the authors describe that deep learning approaches hardly perform better than HMM- or PWM-based prediction methods. However, many studies have shown that machine learning approaches such as CNN or CNN-LSTM models outperform these traditional methods.
- Indeed, several publications focus on Escherichia coli, but many other species have also been subjected to promoter prediction with successful results published.

We appreciate this correction; our original phrasing was indeed too strong and inaccurate. We have revised the introduction to properly acknowledge that deep learning approaches have demonstrated superior performance over traditional methods in benchmark evaluations (lines 40-42).

Additionally, we aimed to more explicitly describe the gap left by current promoter prediction tools. We analysed the annotations of the 3,371 gammaproteobacterial genomes in our training set and found only 263 annotated promoter features compared to nearly 8.5 million annotated coding sequences. We have added this empirical observation to the introduction to demonstrate that despite advances in promoter prediction methodology, systematic genome-wide annotation remains unaddressed (lines 50-54).

We also appreciate the reviewer's point regarding work beyond *E. coli*. We have revised the manuscript to better acknowledge the broader scope of existing studies, adding citations to work on *B. subtilis* and *P. aeruginosa* (lines 45-46)

Results

Recognition of regulatory features
minor
- The authors choose to use 200 nt upstream regions, why no just the intergenic region?

Thank you for raising this point. We have expanded the Methods section to clarify our rationale. The 200 nucleotide window was selected to capture proximal regulatory elements while maintaining high feature density in the training data; longer sequences would dilute the concentration of regulatory motifs. As a secondary benefit, standardising sequence length enabled efficient batch processing with full shuffling during training. We have added this explanation to the Dataset Generation Methods section (lines 359-362).

Whole genome promoter motif annotation
Major
"we manually annotated based on logit plots, consisting of 147 σ70/σ38 promoters, 50 σ54 promoters, 76 σ32 promoters, 27 σ28 promoters, and 18 σ24 promoters." It is unclear on what basis these promoters were selected. Why is this set considered better than known sequences? Moreover, it seems very limited that only 300–400 sequences out of 9 million were chosen to train the model. If these were selected based on knowledge derived from E. coli, this would be a poor choice since E. coli is used as the "leave one out" organism for validation.

We appreciate this concern and have clarified our methodology in the revised manuscript. The 318 sequences represent a transfer learning approach where a lightweight segmentation head is trained atop the frozen base model pretrained on 9 million sequences. This aligns with few-shot learning paradigms in computer vision, where pretrained models achieve strong performance with comparable numbers of task-specific examples. The high validation rates we achieve against ChIP-seq and RegulonDB data (100% for σ24, σ32, σ54; 87% overall precision) demonstrate this approach's effectiveness. In the revised version of the manuscript, we added the RegulonDB validation layer, which substantially improved these numbers compared to the previous submission.

Regarding why existing databases with known sequences were not used: our segmentation task requires box-level annotation, i.e. the precise nucleotide positions of -10/-35 or -24/-12 elements, not merely classifiable promoter sequences. This level of annotation is rare even in well-studied organisms: ChIP-seq identifies binding regions and TSS mapping locates transcription starts, not individually mapped box coordinates. RegulonDB also catalogues promoter regions without systematic box-position annotations. Furthermore, excluding *E. coli* sequences from training was essential to our validation strategy, as *E. coli* is the only organism with sufficient genome-wide experimental data to validate our annotation approach.

Regarding the selection process: training sequences were identified from diverse non-*E. coli* Gammaproteobacterial species by examining upstream regions where logit plots exhibited clear, unambiguous promoter patterns. To ensure sigma factor diversity, we searched for genes from relevant functional categories (e.g., nitrogen utilisation for σ54), then selected sequences with the clearest motif patterns. Promoter element positions were manually annotated based on logit plot peaks, retaining only the most visually obvious examples. While functional categories guided our search for candidate training sequences, the resulting genome-wide predictions identify substantially more promoters than the training set across all species analysed, with GO term enrichment demonstrating biologically coherent patterns across promoter-gene pairs not represented in training. Additional evidence validates this training strategy: predicted promoter distributions deviate from training composition toward biological expectations (12:1 σ70:σ54 in predictions vs. 3:1 in training), indicating genuine pattern recognition rather than memorisation.

We have revised the Methods and Results sections to clarify these points and present the expanded validation results (lines 124-130, lines 147-150, lines 395-409).

This is a strange assumption: “56% for a naive baseline scenario in which σ70 promoters are systematically placed 30 nucleotides upstream of every coding sequence.” This is simply reflecting biological organization — promoter regions are typically located upstream of coding sequences, and most of them are σ70 promoters. The real challenge lies in accurately predicting the Transcription Start Site (TSS) and its associated promoter elements.

We appreciate this feedback. Our intention with this baseline was to demonstrate that existing tools showed only modest improvement over the most naïve baseline assumption. However, we recognise that this comparison is potentially confusing while not providing a substantial contribution to the study. We have removed it from the revised manuscript. The validation framework is now substantially strengthened through the addition of RegulonDB validation and comparison against ProPr54 for σ54 promoters, providing clearer benchmarking that demonstrates PromoterAtlas-annotation's performance advantage over established methods (Figure 3B) without relying on potentially misleading baseline comparisons.

“the smallest amount of promoters predicted in Legionella pneumophila (206), and the largest amount of promoters detected in P. aeruginosa (615).” These numbers are surprisingly low, as several thousand promoters would be expected. P. aeruginosa has 3,932 regions, meaning that a promoter was found in only about 15% of them. Please provide the relevant metrics to clarify this result.

This is a valid concern and we appreciate the reviewer highlighting it. The low numbers reflect our deliberate prioritisation of precision over recall for the main analyses. Following a suggestion from Reviewer 2, we performed a comprehensive sensitivity analysis on segmentation parameters (Supplementary Figure S4), varying the minimum consecutive nucleotides required for annotation (1-6) and toggling co-occurrence enforcement of paired promoter elements. This analysis identified precision-optimised parameters (3 consecutive nucleotides with co-occurrence enforcement) that achieve maximum validation against ChIP-seq data, resulting in conservative annotation of 12-21% of regulatory regions across species (743 promoters in *P. aeruginosa* from 3,673 extracted regions).

However, we recognise that different applications may prioritise sensitivity over specificity. The sensitivity analysis also identified recall-optimised parameters (1 consecutive nucleotide without co-occurrence enforcement) that annotate substantially more promoters. Under these parameters, *P. aeruginosa* shows 2,837 annotated promoters (77% of extracted regions), with similar increases across all analysed species (averaging 70% coverage, Supplementary Figure S6). Both parameter configurations are now available in the annotation tool, allowing users to select settings appropriate for their application.

We have revised the Results section to explicitly clarify that main figures use precision-optimised parameters (lines 143-145, lines 193-198), and we present the recall-optimised results in Supplementary Figure S6 for comparison. The Methods section now details the sensitivity analysis and parameter optimisation strategy (lines 422-432).

Existing programs such as STREME (from the MEME suite) can also identify the same promoter patterns shown here. Does the PromoterAtlas-base model outperform STREME?

We thank the reviewer for this question as it allows us to clarify PromoterAtlas's distinct purpose and the nature of our visualisations.
STREME is a de novo motif discovery tool that identifies statistically enriched patterns from pre-selected sequence sets, outputting position weight matrices with associated p-values. PromoterAtlas serves a different function: it locates and annotates known regulatory elements across complete genomes. These tools address fundamentally different analytical tasks.

The aspect of our work that appears most comparable to STREME would be the logit plots (Figure 2). However, these are qualitative visualisations intended to exploit the pre-trained network in order to reveal the location of regulatory elements in individual sequences, without statistical enrichment scores or PWM outputs. A direct quantitative comparison would therefore not be technically sound or meaningful.

Predicting transcription and translation levels
Looks very promising, with a Pearson correlation of 0.79 on the test set. However, it was somewhat difficult to verify this in detail based on the results provided. This analysis could potentially form an article on its own and may be somewhat beyond the scope of the current manuscript.

We appreciate the reviewer's positive assessment of these results. We agree that verification details were insufficient in the original submission and have addressed this in two ways:

First, we have added Supplementary Table 1 providing comprehensive performance metrics for all transcription and translation prediction models, including Pearson correlations with 95% confidence intervals and evaluations across three expression bins (low, medium, high) to provide performance data across the dynamic range. This table is referenced in lines 259 and 270.

Second, we have made all analysis code publicly available in a second GitHub repository (https://github.com/LucasCoppens/PromoterAtlas_paper) , enabling complete reproduction of the figures and results presented in the manuscript. This is detailed in the code availability section lines 528-529.

Regarding scope: we respectfully maintain that these analyses strengthen rather than distract from the manuscript's central contribution. The key contribution from the expression prediction section is the demonstration that PromoterAtlas embeddings capture functional regulatory information beyond motif identification alone, presenting a key validation that the model learns biologically meaningful representations. This complements the annotation and embedding space analyses by showing the learned features enable quantitative prediction tasks.

Discussion & perspectives

Major
“capture subtle sequence features that influence DNA physical properties such as melting temperature or topological characteristics. These features are typically overlooked by traditional position weight matrices or other methods exclusively focussed on motifs.” This is indeed a limitation of the older PWM-based methods, but such features have been widely incorporated and described in studies employing ML, LLM, or AI models.

We thank the reviewer for this correction. It is true that modern ML approaches have successfully incorporated DNA physical properties, and the original phrasing overstated the novelty of capturing such features. We have substantially revised this paragraph to focus on our approach's true distinguishing characteristics: the data collection strategy of training on millions of upstream sequences across thousands of species within a phylogenetic clade through unsupervised pretraining (lines 294-306). This middle-ground approach sits somewhere between small experimentally validated datasets and broad genomic foundation models, allowing it to exploit evolutionary conservation whilst maintaining task-oriented focus. The revised text now uses more measured language regarding learned representations ("without requiring explicit feature engineering" rather than claiming to capture specific physical properties), appropriately acknowledging that the nature of learned features remains an empirical question rather than a demonstrated novelty in our study.

“Current expression prediction tools for bacterial systems are primarily developed for and validated in E. coli” and other model organisms.

We have directly incorporated this suggestion (lines 323-324), which appropriately broadens the statement to acknowledge that expression prediction tools extend beyond *E. coli to* include other model organisms.

Software
minor
- The software was easy to install and ran smoothly. The results are provided only in GenBank format; adding support for additional formats would be useful to enable import into other visualization or analysis packages.

We thank the reviewer for this suggestion and have added GFF3 and FASTA format support in addition to GenBank (lines 523-527 in Code availability, updated in GitHub repository). GFF3 support had also been independently requested by a user in our GitHub repository (issue #1), confirming the community need for broader format compatibility. The updated software now supports GenBank, GFF3, and FASTA formats, facilitating integration with diverse analysis and visualisation tools. GenBank and GFF3 inputs are converted to GenBank and GFF3 outputs, while FASTA results are presented in tsv file format.

Reviewer #1 (Remarks on code availability):

The software tool is easy to install, is working good, and is user friendly. The output is too limited and additional formats should be added.

Please see our response to the Software section above regarding GFF3 format support.

Reviewer #2 (Remarks to the Author):

In this manuscript, the authors presented PromoterAtlas, a transformer-based model trained on 9 million regulatory sequences for the identification and annotation of bacterial promoters. The authors demonstrate the model's utility in multiple tasks, including the visualization of regulatory features, genome-wide annotation of promoters for five σ factors, prediction of gene expression levels, and capturing evolutionary relationships in its embedding space. The scale of the pre-training data and the integration of these diverse capabilities into a single, end-to-end framework is a significant strength. The work addresses a important challenge in bacterial genomics. The work is technically sound and describes a significant methodological advancement in the field of computational genomics. However, some major points need to be addressed.

1.The study presents several intriguing observations, such as the encoding mechanisms for σ28 promoters and the phylogenetic relationships captured in the embedding space. However, the current analysis primarily describes these patterns without sufficiently investigating their underlying biological causes or functional consequences. To strengthen the study and move beyond correlation towards causation, it is recommended that the authors attempt to validate a key prediction by biological experiments.

We appreciate the reviewer's interest in these findings. While experimental validation would be valuable future work, the current study provides substantial computational validation through multiple independent approaches: ChIP-seq combined with the newly added RegulonDB validation achieving 100% precision for three sigma factors, GO enrichment demonstrating functional coherence across 13 species, and expression predictions reaching state-of-the-art Pearson correlations. These convergent lines of evidence directly demonstrate the validity of the methods presented.

Regarding the embedding space analysis: we present this as a novel approach to interpreting learned representations in DNA sequence models, not as a biological discovery claiming causation. To our knowledge, this represents the first systematic analysis of its kind characterising how a transformer network progressively organises DNA regulatory sequence information across its layers. We have revised the section title and text (lines 209, 211, 225) to frame these observations more carefully as characterisation of the model's computational representations rather than claims about evolutionary mechanisms. The analysis complements the practical annotation capabilities by providing interpretable insight into what the model learns, which we consider appropriate for a computational methods paper focused on developing scalable analysis tools.

2.The manuscript currently states that sequences upstream of coding regions were extracted but lacks critical methodological details. Specifically, the procedure for handling the directional nature of DNA strands (e.g., how upstream regions were defined and extracted for DNA sequences containing both positive and negative strands) is not explained. A more comprehensive description of the data processing pipeline is essential to ensure the reproducibility of the study.

We thank the reviewer for identifying this gap in our methodological description. We have added clarification to the Dataset Generation section (lines 362-364) specifying that for sequences on the negative strand, the reverse complement was extracted such that all sequences represent the 5' to 3' coding strand orientation. The full data processing pipeline is now also available in the additional github repository with scripts and notebooks aimed at full reproducibility of any data collection, results, and figures in our study: https://github.com/LucasCoppens/PromoterAtlas_paper.

3.The PromoterAtlas-base model was pre-trained on 200-nucleotide sequences, yet the transcription and translation prediction models were trained and evaluated on sequences standardized to a length of only 86 nucleotides. The authors do not explain the rationale for this significant discrepancy in input length. A detailed justification for this specific length choice and a discussion of its potential impact on model performance and biological relevance are necessary.

We thank the reviewer for highlighting this point. The 86-nucleotide length reflects the experimental datasets themselves rather than a design choice: this is the length of the longest sequence across all four transcription prediction datasets, which derive from synthetic promoter libraries in the referenced studies. We have clarified this in the Methods section (lines 471-472). The strong performance achieved demonstrates that the model effectively extracts regulatory information from these sequences despite the length discrepancy with pretraining data.

4. The threshold for the "minimum of 4 consecutive nucleotides" in the genome annotation pipeline seems arbitrary. Was this parameter optimized? Please provide a justification or a sensitivity analysis.

We thank the reviewer for this excellent suggestion. We have performed a comprehensive sensitivity analysis on segmentation parameters, varying the minimum consecutive nucleotide threshold from 1 to 6 and toggling co-occurrence enforcement of paired promoter elements (Supplementary Figure S4). This analysis evaluated precision and recall scores against the *E. coli* ChIP-seq validation data across all parameter combinations.

The analysis identified a threshold of 3 consecutive nucleotides with co-occurrence enforcement as optimal for maximizing precision, which we adopted for the main figures. We have also characterized recall-optimized parameters (1 consecutive nucleotide without co-occurrence enforcement) that substantially increase coverage by prioritising sensitivity over specificity, presented in Supplementary Figure S6. Both parameter configurations are now available in the annotation tool, allowing users to select settings appropriate for their applications.

We have revised the Results and Methods sections (lines 136-145, lines 193-198, lines 422-432) to detail this optimisation procedure. The main figures present results using precision-optimised parameters, with recall-optimised annotation results provided in Supplementary Figure S6 for comparison.

5.The manuscript suffers from substantial textual overlap between the Methods and Results sections. For instance, the detailed description of the sequence standardization process and the architecture of the prediction models in the "Prediction of transcription levels" (Methods) is virtually identical to the text in the "Predicting transcription and translation levels" (Results). This extensive repetition, from "All sequences were standardised to a maximum length of 86 nucleotides..." to "...but operated on raw sequence one-hot encodings rather than learned embeddings," undermines the manuscript's professionalism. The Results section should focus on presenting and interpreting the findings (e.g., performance metrics, correlation values), not on reiterating methodological details. The Methods section exists to provide the comprehensive technical description required for reproducibility. I strongly recommend that the authors thoroughly revise the entire manuscript to eliminate these redundancies. The Results section should present a clear narrative of the discoveries, while the Methods section should be preserved as the single, authoritative source for the experimental and computational procedures.

We thank the reviewer for this important criticism. The reviewer is correct that the original manuscript contained substantial overlap between Results and Methods sections. We have thoroughly revised the entire manuscript to eliminate these redundancies. The Results section now focuses on presenting findings and their biological interpretation, while all methodological details have been consolidated in the Methods section as the single authoritative source for reproducibility. We have also rewritten parts of the discussion section as part of this restructuring. This revision has substantially improved the manuscript's clarity and structure.

6.The authors should more clearly articulate the specific advantages of their PromoterAtlas approach when compared to contemporary general-purpose genomic foundation models, such as Evo and Evo2. The manuscript currently lacks a direct performance comparison with these state-of-the-art models on relevant tasks (e.g., promoter annotation or expression prediction). Given that these models are trained on trillions of nucleotides across diverse species and have demonstrated powerful capabilities in functional element prediction, it is essential to benchmark PromoterAtlas against them. he authors should discuss their method's comparative performance and justify its value.

We thank the reviewer for this important suggestion. We have added comprehensive benchmarking against Evo2 for transcription prediction tasks. We followed the expression prediction methodology established in the original Evo publication, applied to Evo2 (which the authors report performs as well as Evo1 on bacterial tasks). Compared to annotation, expression prediction provided the more rigorous comparison point: multiple independent datasets with quantitative ground truth enable systematic cross-dataset evaluation, and following the established Evo evaluation framework ensures methodological fairness.

PromoterAtlas-based prediction models outperformed Evo2-based models across the datasets, with detailed performance metrics available in Supplementary Table 1. We suggest that this comparison demonstrates a key advantage of domain-specific pretraining: PromoterAtlas embeddings, despite being approximately 4000 times smaller than Evo2 (1.8M vs 7B parameters), enable higher performance on regulatory sequence tasks through focused training on bacterial regulatory regions.

We have revised the Results, Discussion, and Methods sections (lines 250-264, lines 333-340, lines 474-487) to present these comparisons and contextualise the implications of domain-specific versus general-purpose model architectures for regulatory sequence analysis.
